# Su-Schrieffer-Heeger chain with one pair of $\mathcal{P}\mathcal{T}$-symmetric defects

**DOI:** 10.1038/s41598-017-06198-9

**Published:** 2017-07-19

**Authors:** L. Jin, P. Wang, Z. Song

**Affiliations:** Nankai University, School of Physics, Tianjin, 300071 P. R. China

## Abstract

The topologically nontrivial edge states induce $\mathcal{P}\mathcal{T}$ transition in Su-Schrieffer-Heeger (SSH) chain with one pair of gain and loss at boundaries. In this study, we investigated a pair of $\mathcal{P}\mathcal{T}$-symmetric defects located inside the SSH chain, in particular, the defects locations are at the chain centre. The $\mathcal{P}\mathcal{T}$ symmetry breaking of the bound states leads to the $\mathcal{P}\mathcal{T}$ transition, the $\mathcal{P}\mathcal{T}$-symmetric phases and the localized states were studied. In the broken $\mathcal{P}\mathcal{T}$-symmetric phase, all energy levels break simultaneously in topologically trivial phase; however, two edge states in topologically nontrivial phase are free from the influence of the $\mathcal{P}\mathcal{T}$-symmetric defects. We discovered $\mathcal{P}\mathcal{T}$-symmetric bound states induced by the $\mathcal{P}\mathcal{T}$-symmetric local defects at the SSH chain centre. The $\mathcal{P}\mathcal{T}$-symmetric bound states significantly increase the $\mathcal{P}\mathcal{T}$ transition threshold and coalesce to the topologically protected zero mode with vanishing probabilities on every other site of the left-half chain and the right-half chain, respectively.

## Introduction

The parity-time ($\mathcal{P}\mathcal{T}$) symmetric non-Hermitian Hamiltonians can possess real spectra^1–8^ but nonunitary dynamics, such as faster evolution^9, 10^ and power oscillation^11^. The $\mathcal{P}\mathcal{T}$ system experiences a phase transition when its spectrum changes between real and complex. The $\mathcal{P}\mathcal{T}$ transition point is the exceptional point associated with eigenstates coalescence^8^. In one-dimensional system, the exceptional point varies as system size and structure^12, 13^. The critical gain/loss rate approximately equals to the coupling strength of a uniform chain for a pair of $\mathcal{P}\mathcal{T}$-symmetric gain and loss defects at boundary^14^. For defects locations at center, the critical gain/loss rate is the coupling strength between the two defects^15^. Topological properties were extensively investigated in condensed matter physics^16–27^ and in photonic systems^28–30^; furthermore, the $\mathcal{P}\mathcal{T}$-symmetric topological insulator was proposed in two-dimensional (2D) coupled optical resonators. Different with traditional Hermitian topological insulator, the edge states are unidirectional amplified and damped^31^. Topological insulator states are $\mathcal{P}\mathcal{T}$ symmetry breaking in a $\mathcal{P}\mathcal{T}$-symmetric non-Hermitian extension of Dirac Hamiltonians, because the $\mathcal{P}\mathcal{T}$ operator switches the edge states locations at boundary^32^. The Chern number was generalized for non-Hermitian systems, the tight-binding model on the honeycomb and square lattices under different symmetry classes were examined, broken $\mathcal{P}\mathcal{T}$-symmetric edge states with real part eigen energies being zero were found^33^. The topologically chiral edge modes found in 2D non-Hermitian system were related to the exceptional point of the bulk Hamiltonian that characterized by two half-integer charges of the exceptional point^34^.

The Su-Schrieffer-Heeger (SSH) chain^35^ with a pair of $\mathcal{P}\mathcal{T}$-symmetric defects at boundary was studied, the edge states found in topologically nontrivial phase are sensitive to the non-Hermiticity^36^ and the critical non-Hermitian gain and loss approach zero^37^. The non-Hermitian Kitaev and extended Kitaev models were investigated similar as the SSH model^38, 39^. Optical systems are fruitful platforms for the investigation of $\mathcal{P}\mathcal{T}$ symmetry^40–46^. The robust light interface states were discovered at the interface of a combined two SSH chains with different quantum phases of $\mathcal{P}\mathcal{T}$ symmetry^47^. Recently, non-Hermitian SSH chains were experimentally realized by coupled dielectric microwave resonators^48, 49^ and photonic lattices^50, 51^. In passive SSH chains with periodical losses, single coupling disorder induces asymmetric topological zero mode^49^ and $\mathcal{P}\mathcal{T}$-symmetric topological zero mode interface states, respectively^51^. $\mathcal{P}\mathcal{T}$ symmetry breaking and topological properties were theoretically investigated in other $\mathcal{P}\mathcal{T}$-symmetric systems^52–54^, the competition between two lattice defects can induce $\mathcal{P}\mathcal{T}$ symmetry breaking and restoration as non-Hermiticity increasing in Aubry-André-Harper model^55^.

In this work, we study an open SSH chain with one pair of $\mathcal{P}\mathcal{T}$-symmetric gain and loss. The $\mathcal{P}\mathcal{T}$-symmetric thresholds, the topologically nontrivial edge states, and the local defects induced $\mathcal{P}\mathcal{T}$-symmetric bound states are investigated. The $\mathcal{P}\mathcal{T}$-symmetry breaking is closely related to the appearance of localized states. When considering the defects located near the chain boundary, the edge states in topologically nontrivial region break the $\mathcal{P}\mathcal{T}$ symmetry if the defects are at the sites with nonzero states distribution probabilities; otherwise, the edge states are free from the influence of the on-site defects and the $\mathcal{P}\mathcal{T}$ symmetry phase transition is induced by the bulk states: The extended (bound) state induces the $\mathcal{P}\mathcal{T}$ symmetry phase transition at weak (strong) non-Hermiticity for defects near the chain boundary (at the chain center).

The $\mathcal{P}\mathcal{T}$ transition threshold is the largest when the defects located at the chain center, being the weak inhomogeneous couplings of the SSH chain. Two edge states and four bound states exist at large non-Hermiticity, the number of breaking energy levels increases as defects moving from the chain boundary to the center. For defects near the chain center, when $\mathcal{P}\mathcal{T}$ transition happens, all energy levels break simultaneously in topologically trivial phase; by contrast, two topologically nontrivial edge states are not $\mathcal{P}\mathcal{T}$ symmetry breaking although in broken $\mathcal{P}\mathcal{T}$ symmetry phase. The $\mathcal{P}\mathcal{T}$ transition is associated with the $\mathcal{P}\mathcal{T}$ symmetry breaking of the eigenstate. The edge states and bound states probabilities localize around the $\mathcal{P}\mathcal{T}$-symmetric defects; therefore, they are $\mathcal{P}\mathcal{T}$ symmetry breaking states except when the defects are the nearest neighbours. We discovered a pair of $\mathcal{P}\mathcal{T}$-symmetric bound states for the defects at the chain centre, the $\mathcal{P}\mathcal{T}$-symmetric bound states significantly increase the $\mathcal{P}\mathcal{T}$ transition threshold, at which, the bound states coalesce to the topologically protected zero mode but their probabilities are not only confined to either the loss or the gain sublattice; the probabilities vanish on every other site of the left-half chain and the right-half chain, respectively.

## Results

### $\mathcal{P}\mathcal{T}$-symmetric non-Hermitian SSH chain

**Figure 1:**
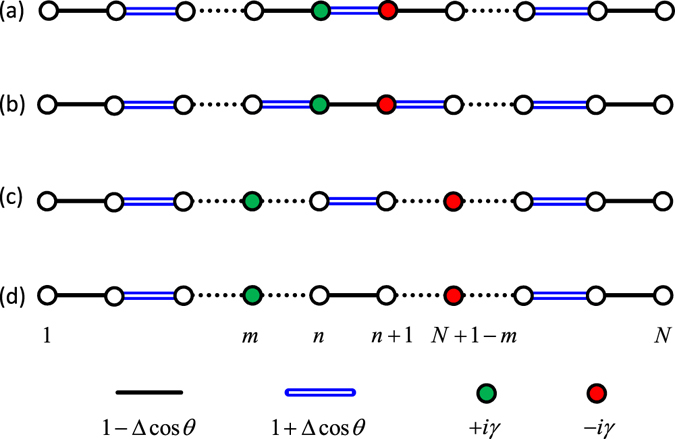
Schematic illustration of the $\mathcal{P}\mathcal{T}$-symmetric SSH chain. The total chain number is even *N* = 2*n*. The balanced gain and loss are at site *m* and its $\mathcal{P}$-symmetric site *N* + 1 − *m*. (**a**) Even *n*, *m* = *n*, (**b**) odd *n*, *m* = *n*, (**c**) even *n*, (**d**) odd *n*. The red and blue lines represent the inhomogeneous couplings. The gain and loss sites are in green and red, respectively.

In this section, we introduce a one dimensional *N*-site SSH chain with one pair of $\mathcal{P}\mathcal{T}$-symmetric imaginary defects, the system is schematically illustrated in Fig. 1. The couplings between neighbor sites are staggered 1 ± Δcos*θ*, which are modulated by parameter Δ. The coupled chain can be realized by optical resonators^56–58^. The defects pair includes a loss (in red) and a balanced gain (in green)^5, 6, 11, 40, 42, 45^. We define $\mathcal{P}$ as the parity operator, which equals to a reflection symmetry with respect to the chain center, satisfying $\mathcal{P}j{\mathcal{P}}^{-1}=N+1\,-\,j$. The time-reversal operator satisfies $\mathcal{T}i{\mathcal{T}}^{-1}=-i$. Under these definitions, the balanced gain and loss as on-site defects pair satisfies the $\mathcal{P}\mathcal{T}$ symmetry. The primary SSH Hamiltonian *H*_0_ is in form of ${H}_{0}={\sum }_{j}^{N-1}[1+{(-1)}^{j}{\rm{\Delta }}\,cos\,\theta ]({c}_{j}^{\dagger }{c}_{j+1}+{c}_{j+1}^{\dagger }{c}_{j})$, where ${c}_{j}^{\dagger }$ (*c*_*j*_) is the creation (annihilation) operator on site *j* for fermionic particles. The chiral symmetry protects the topological properties of *H*_0_ in topologically nontrivial region. In this work, we confine our discussion within system with even *N*, the non-Hermitian gain and loss defects are located at reflection symmetric positions *m* and *N* + 1 − *m*; the non-Hermitian extended SSH chain is

1 $$H={H}_{0}+i\gamma {c}_{m}^{\dagger }{c}_{m}\,-\,i\gamma {c}_{N+1-m}^{\dagger }{c}_{N+1-m}.$$

Note that *H* satisfies the $\mathcal{P}\mathcal{T}$ symmetry, and is expected to have purely real spectrum. The analysis and conclusions are applicable to corresponding bosonic particles^59^.

Topologically nontrivial edge states disappear in system with universal non-Hermiticity^32, 33^, but remain in system with localized non-Hermiticity^36, 53^. *The topology is changed in the presence of universal non-Hermiticity, but is robust to several impurities although the impurities are non-Hermitian*. The traditional Hermitian SSH model has inhomogeneous staggered hoppings between neighbor sites, the SSH Hamiltonian is a two-band model. Under periodical boundary condition, the Berry phases of the two bands can be calculated, both being *π* in topologically nontrivial phase when −*π*/2 < *θ* < *π*/2 and both being 0 in topologically trivial phase when −*π* ≤ *θ* ≤ −*π*/2 and *π*/2 ≤ *θ* ≤ *π*. Under open boundary condition, the bulk-edge correspondence indicates the existence of edges states. For even *N*, two zero edge states appear in region −*π*/2 < *θ* < *π*/2; by contrast, for odd *N*, single edge state exists when *θ* ≠ ±*π*/2^59^. The edge states probabilities are localized on the chain boundary, *thus edge states are immune to non-Hermitian on-site defects at the SSH chain center*.

### $\mathcal{P}\mathcal{T}$-symmetric phases

**Figure 2:**
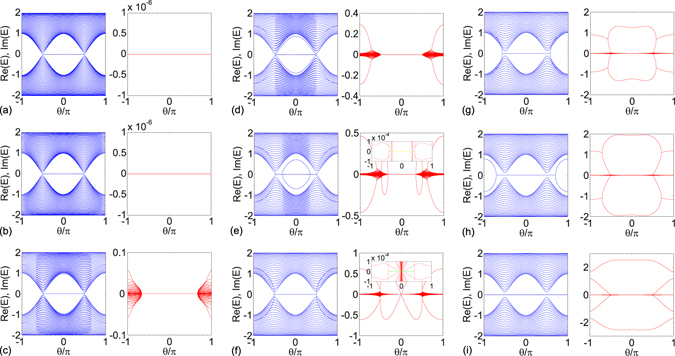
Spectrum under open boundary condition as a function of *θ*. (**a**) *γ* = 1/4, (**b**) *γ* = 1/2, (**c**) *γ* = 3/4, (**d**) *γ* = 1, (**e**) *γ* = 5/4, (**f**) *γ* = 3/2, (**g**) *γ* = 2, (**h**) *γ* = 5/2, (**i**) *γ* = 3. Other system parameters are *N* = 100, Δ = 1/2; the gain and loss are at sites 50 and 51.

We first consider $\mathcal{P}\mathcal{T}$-symmetric SSH chain with even *n* as follows (Fig. 1a). The defects are at the chain center (*m* = *n*). The SSH chain with odd *n* has similar results (Fig. 1b). The Hamiltonian *H* is always in the exact $\mathcal{P}\mathcal{T}$-symmetric phase as *θ* varies when *γ* < 1 − Δ; the exact $\mathcal{P}\mathcal{T}$-symmetric region shrinks for 1 − Δ < *γ* < 1 + Δ. At *γ* > 1 + Δ, *H* is in the broken $\mathcal{P}\mathcal{T}$-symmetric phase for arbitrary *θ*. In $\mathcal{P}\mathcal{T}$ symmetry broken phase, all energy levels are $\mathcal{P}\mathcal{T}$ symmetry breaking in topologically trivial region; however, two edge states are robust to gain and loss defects in topologically nontrivial region, and can be composed into a pair of $\mathcal{P}\mathcal{T}$-symmetric states. As an illustration, we numerically calculate the SSH chain spectra and depict them as a function of *θ* for *N* = 100 and Δ = 1/2 in Fig. 2. The real and imaginary parts of the spectra are plotted in blue and red lines, respectively. Two topologically nontrivial edge states appear in the region −*π*/2 < *θ* < *π*/2.

Figure 2a,b show the entirely real spectrum as a function of *θ* at *γ* = 1/4 and 1/2; however, the $\mathcal{P}\mathcal{T}$-symmetric SSH chain is nondiagonalizable for *γ* = 1/2 at *θ* = ±*π*, where the coupling inhomogeneity is at maximum. The Hamiltonian *H* become *n* 2 × 2 Jordan blocks after diagonalization, which indicates *n* pairs of two states coalescence. For *γ* > 1/2, the $\mathcal{P}\mathcal{T}$ symmetry breaking appears at *θ* = ±*π*, the $\mathcal{P}\mathcal{T}$ symmetry of all eigen states breaks simultaneously. In Fig. 2c, we depict the SSH chain spectrum for *γ* = 3/4. The exact $\mathcal{P}\mathcal{T}$-symmetric region is determined by *γ* = 1 +Δcos*θ*. Thus, the system is in exact $\mathcal{P}\mathcal{T}$-symmetric phase in region −2*π*/3 ≤ *θ* ≤ 2*π*/3; in other regions, all the *N* eigenvalues form *n* conjugate pairs. The eigen states with the largest imaginary part (we refer to the absolute values in the comparison) have highest probabilities localized near the chain center. The inverse participation ratio (sum of fourth power of the wave function amplitude ∑_*j*_|*ψ*_*j*_|^4^) for the extended state scales as system size in order of *N* ^−1^, but the IPR for the localized state approaches constant at large system size. As *γ* increasing, the probabilities are more localized and form bound states at approximately *γ* ≳ 1. The bound states are attributed to the $\mathcal{P}\mathcal{T}$-symmetric non-Hermitian impurities; and the bound states probabilities are localized near the chain center.

In Fig. 2d, *γ* = 1, exact $\mathcal{P}\mathcal{T}$-symmetric phase shrinks to −*π*/2 ≤ *θ* ≤ π /2. In broken $\mathcal{P}\mathcal{T}$-symmetric phase, the two red lines (two folders) with the maximum imaginary parts in regions −*π* ≤ *θ* ≲ −0.7*π* and 0.7*π* ≲ *θ* ≤ *π* correspond to four bound states. Notably, the bound states eigenvalues change more rapidly than the extended states and enter the extended states band around *θ* ≈ ±0.7*π*. As *γ* increasing to *γ* = 5/4 as shown in Fig. 2e, exact $\mathcal{P}\mathcal{T}$-symmetric phase shrinks to −*π*/3 ≤ *θ* ≤ *π*/3 (indicated by yellow line in the inset), where the SSH chain has two degenerate zero edge states and two bound states with real eigenvalues. The $\mathcal{P}\mathcal{T}$ symmetry breaks out of region −*π*/3 ≤ *θ* ≤ *π*/3. In the regions −*π*/2 ≲ *θ* < −*π*/3 and *π*/3 < *θ* ≲ *π*/2, the SSH chain is in broken $\mathcal{P}\mathcal{T}$-symmetric phase with two topologically nontrivial edge states. There exist two bound states and two edge states, the two bound states have pure imaginary eigenvalues with the largest imaginary part, exiting the extended states band at *θ* ≈ ±*π*/2 and become real for |*θ*| ≤ *π*/3. Notably, the two edge states are also pure imaginary but with the smallest imaginary parts, approaching zero as |*θ*| close to 0. This is because weak inhomogeneity for |*θ*| close to *π*/2 induces more spreading of edges states; by contrast, strong inhomogeneity for |*θ*| close to 0 induces more localization of the edge states. In the numerical results, the imaginary parts of edge states are negligible in the region −0.4*π* ≲ *θ* ≲ 0.4*π* (indicated by green line in the inset), determined by the coupling inhomogeneity Δ. *The two zero edge states are free from the influence of non-Hermitian defects at (or close to) the chain center*. In the regions −*π* ≤ *θ* ≤ −*π*/2 and *π*/2 ≤ *θ* ≤ *π*, there are four bound states with the largest imaginary eigenvalues. In Fig. 2f, the spectrum for *γ* = 3/2 is plotted. The bound states always have the largest imaginary eigenvalues, the bound states are two folders out of region −*π*/2 < *θ* < *π*/2, and one folder in region −*π*/2 < *θ* < *π*/2. For *γ* > 3/2, the SSH chain spectrum is in the broken $\mathcal{P}\mathcal{T}$-symmetric phase at arbitrary *θ*. *The only real energy states are the two topologically nontrivial edge states in the region* −0.4*π* ≲ *θ* ≲ 0.4*π* (indicated by green line in the inset).

In Fig. 2g,f, 3/2 < *γ* < 2.96, the imaginary parts of the bound states experience a bifurcation in topological trivial regions −*π* ≤ *θ* ≤ −*π*/2 and *π*/2 ≤ *θ* ≤ *π*. After bifurcation, the eigenvalues of the bound states become pure imaginary, which is reflected from the real parts being zero. In the real part of the energy spectrum, the zero is four folders, and corresponds to the four bound states in the topologically trivial phase but corresponds to two bound states and two edge states in the topologically nontrivial phase. Notably, the four folders zero always exists in the real part of energy spectrum for *γ* > 2.96. The bound states have pure imaginary eigenvalues. The bifurcation behavior disappears in the imaginary parts of the bound states. In this situation, one pair of bound states have larger imaginary eigenvalues than the other pair at arbitrary *θ*.

In the situation of odd *n* (Fig. 1b), the coupling between the gain and loss is 1 − Δcos*θ*; by contrast, this coupling is 1 + Δcos*θ* for even *n* case. The spectrum structures are approximately the same as the even *n* case shown in Fig. 2, but shifted by *π* in parameter *θ*, but two zero edge states still exist in topologically nontrivial phase −*π*/2 < *θ* < *π*/2. In our discussion of odd *n* case, the coupling at the boundaries are unchanged as 1 − Δcos*θ*.

In the following, we discuss a general situation that the $\mathcal{P}\mathcal{T}$-symmetric defects are inside the SSH chain (0 < *m* < *n*) rather than at the SSH chain center. The configuration is illustrated in Fig. 1c,d. The topological properties are robust to the one pair of gain and loss defects; however, the $\mathcal{P}\mathcal{T}$-symmetric properties change significantly. The edge states with probabilities localized at the chain boundary are free from the influence of non-Hermitian defects when they are close to the chain center (*m* ~ *n*) at strong coupling inhomogeneity. By contrast, for the gain and loss defects at the chain boundary (*m* = 1), the $\mathcal{P}\mathcal{T}$ symmetry of the SSH chain is fragile to the non-Hermitian defects in the presence of topologically nontrivial edge states. This is because the probabilities of edge states are the highest at the chain boundary and decay exponentially. Thus, the influence of defects is the greatest for edge states; any small gain and loss rate breaks the $\mathcal{P}\mathcal{T}$ symmetry of the SSH chain in the topologically nontrivial region −*π*/2 < *θ* < *π*/2; in topologically nontrivial region, the two edge states, forming a conjugation pair with pure imaginary eigenvalues, are the only $\mathcal{P}\mathcal{T}$ symmetry breaking states^36^.

As parameter *θ* varies, the number of breaking energy levels at maximum appears in the topologically trivial phase. The number of breaking energy levels at maximum is larger for larger *m*, which equals to 2 *m* + 2 for odd *m* ≤ *n* − 2 in the topologically trivial phase but equals to 2 *m* in the topologically nontrivial phase. By contrast, the number of breaking energy levels at maximum is 2 *m* for *m* = *n* − 1 and *n* in both topologically trivial and nontrivial phases. When *m* = *n*, all energy levels break simultaneously as shown in Fig. 2c–e. *The number of breaking levels at maximum in topologically nontrivial region is two less than that in topologically trivial region for m* ≤ *n* − 2 *at odd m*.

For *N* = 100, in topologically nontrivial phase of *θ* = 0, the coupling inhomogeneity is the strongest. The number of $\mathcal{P}\mathcal{T}$-symmetric breaking energy levels at maximum is 2 *m* for even *m* < *n* − 2; which changes to 2 *m* − 2 (2 *m*) for odd 25 ≤ *m* < *n* − 2 (*m* < 25). The two edges states have pure imaginary eigenvalue |*E*_ES_| < 10^−10^ for *m* ≥ 25 along with defects moving from the chain boundary to the center, the edge states can be considered as unaffected and the eigenvalues are zero for odd *m* ≥ 25. The critical gain/loss rate is *γ*_c_ ≈ 0.07 at *m* = 25. At weak non-Hermiticity ($\gamma \ll 1$), bound states disappear and two pairs (four) of extended states break first with equal amount of energy imaginary parts. For *n* − 2 ≤ *m* ≤ *n*, the number of $\mathcal{P}\mathcal{T}$-symmetric breaking energy levels at maximum is 2 *m* − 2. The topologically nontrivial zero edge states are real valued.

### Edge states and bound states

The defects support localized modes, which can induce $\mathcal{P}\mathcal{T}$ symmetry breaking^60^. In the SSH chain, the edge states break the $\mathcal{P}\mathcal{T}$ symmetry if the gain and loss are at the sites with nonzero distribution probabilities in topologically nontrivial phase; otherwise, the edge states are free from the influence of gain and loss and the $\mathcal{P}\mathcal{T}$ symmetry phase transition is induced by the bulk states, including the extended states induced $\mathcal{P}\mathcal{T}$ symmetry phase transition at weak non-Hermiticity for defects near the chain boundary and the bound states induced $\mathcal{P}\mathcal{T}$ symmetry phase transition at strong non-Hermiticity for defects at the chain center.

**Figure 3:**
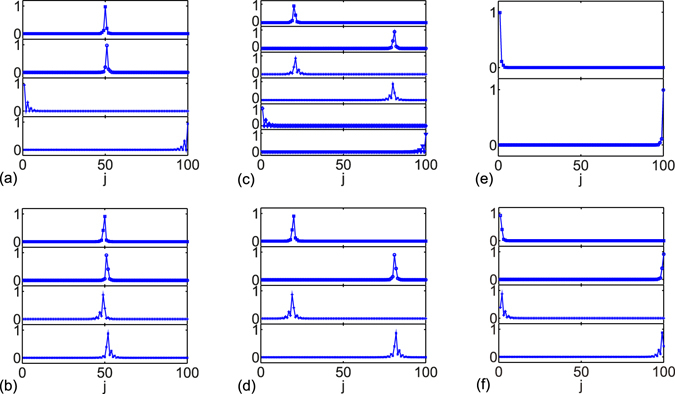
The localized edges states and bound states. (**a**,**b**) *m* = 50, (**c**,**d**) *m* = 20, (**e**,**f**) *m* = 1. In the plots, *θ* = 0 in (**a**,**c**,**e**), *θ* = *π* in (**b**,**d**,**f**). Other parameters are *γ* = 4, *N* = 100, Δ = 1/2.

The probabilities of two edge states for *γ* = 0 are staggered decreasing from the chain boundary^59^, the probability approaches zero for every other site. The probabilities of two edge states on site *m* and its $\mathcal{P}$-symmetric position *N* + 1 − *m* are both zeros for even *m*. Thus, the edge states are unaffected (Fig. 3a,c). By contrast, the influence of defects pair on the edge states is remarkable for odd *m*, in particular, for the defects close to the chain boundary (Fig. 3e). The topologically nontrivial edge states are robust to non-Hermitian defects, the nonvanishing distribution probabilities at the chain boundary lead to $\mathcal{P}\mathcal{T}$ symmetry breaking, and the energies of two edge states become conjugate imaginary pair for small gain and loss^37^.

At large non-Hermiticity, six localized states at maximum are found, which include four bound states and two edge states as depicted in Fig. 3c. The number of localized states depends on the non-Hermiticity and the defects locations. For large enough gain and loss, all localized states eigenvalues have zero real parts. In topologically trivial phase, the localized states are four bound states; however, the number differs in topologically nontrivial phase. Figure 3 depicts the edge states and bound states probabilities for different defects locations. For *m* = 1, the defects at the chain boundary, two edge states are the only localized states; for 2 ≤ *m* ≤ *n* − 1, two edge states and four bound states are found; for *m* = *n*, there are four localized states, including two edge states and two bound states.

In Fig. 3a, the localized states in topologically nontrivial phase with gain and loss at the center are depicted. The localized states include one conjugate pair of bound states and two edge states. The eigenvalues are +3.6494*i*, −3.6494*i*, and two degenerate 0 from top to bottom. In Fig. 3b, the SSH chain is in topologically trivial phase, the eigenvalues of two pairs of bound states are +3.2995*i*, −3.2995*i*, +0.6062*i*, and −0.6062*i* from the top to the bottom.

In Fig. 3c, the eigenvalues of the four bound states are +3.2594*i*, −3.2594*i*, +0.6136*i*, and −0.6136*i* from top to middle. The other two states at bottom are the zero edge states. In Fig. 3d, the eigenvalues of four bound states are the same as those shown in Fig. 3c, but with probabilities distributions slightly different. This is because *θ* = 0 in Fig. 3c but *θ* = *π* in Fig. 3d, the two couplings 1 ± Δ between each site and its nearest neighbors switch in the two structures. The bound states probabilities are localized near the defects and decay to zero at the chain boundary. Therefore, the SSH chain structures at the boundary are not important for the four bound states and they are approximately identical in the two cases of *θ* = 0 and *θ* = *π*. This conclusion is invalid when the gain and loss defects locations are close, where bound states probabilities decay from defects *iγ* and −*iγ* affected each other.

In case of defects at the chain boundary, the bound states disappear in topologically nontrivial phase. In Fig. 3e, the two edge states eigenvalues are +3.9445*i*, −3.9445*i*, and being ±*iγ* for *n* → ∞, both two edge states are fragile to impurities at the chain boundary. The staggered decay of edge states disappears when *γ* is large. In Fig. 3f, the eigenvalues are +3.3384*i*, −3.3384*i*, +0.5991*i*, and −0.5991*i*. The staggered decay of state probabilities is clearly seen for states with small imaginary eigenvalues (absolute values).

The bound states with positive (negative) imaginary eigenvalues are centered at the gain (loss) site. In the two pairs of bound states, the probabilities decay faster for the one with larger imaginary eigenvalues. The probabilities maxima of these two bound states are at the gain and loss sites. The other pair of bound states has smaller imaginary eigenvalues, which decay in a staggered way instead of monotonously and the decay is slower. The probabilities maxima of this pair of bound states are at the nearest neighbor site of the impurity, the site which has stronger coupling strength between the impurity and its neighbors. As shown in Fig. 3c,d, the stronger couplings are 1 + Δ between 20 (81) and 21 (80) as shown in Fig. 3c and 19 (82) and 20 (81) as shown in Fig. 3d. The pair of bound states with smaller positive (negative) imaginary parts are localized at 21 (80) and 19 (82), respectively. By contrast, for the gain and loss at the chain center, bound states with smaller imaginary eigenvalues vanish (Fig. 3a). The situations are different for defects at the boundary (*m* = 1) and the center (*m* = 50), the dimerized unit with stronger couplings is incomplete at the boundary and the center in comparison with other cases, and the localized states partially vanish accordingly.

**Figure 4:**
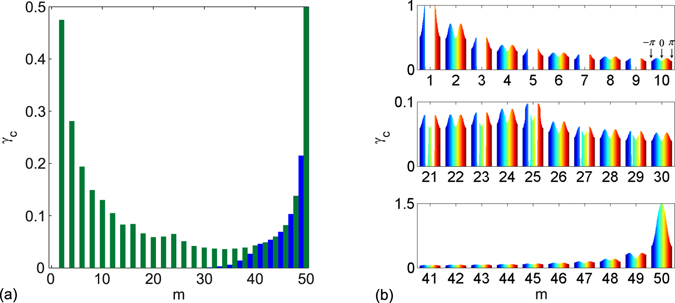
The numerically calculated *γ*_c_ as a function of location *m*. (**a**) *γ*_c_ minimum in region *θ* ∈ [−*π*, *π*] is depicted, which indicates the $\mathcal{P}\mathcal{T}$-symmetric SSH chain spectrum is entirely real for *γ* < *γ*_c_ at arbitrary *θ*. The blue bars are for odd *m*, which approaches zero for *m* < 30; the green bars are for even *m*. (**b**) *γ*_c_ depicted for full *θ* ∈ [−*π*, *π*], the dark blue from left to dark red on the right represent *θ* from −*π* to *π*, the green area in the center corresponds to *θ* = 0, indicated by arrows in the upper right corner. Other parameters are *N* = 100, Δ = 1/2.

In Fig. 4a, we depict *γ*_c_ as a function of location *m*. *γ*_c_ is maximal at *m* = *n*, being 1 − Δ; the minimum *γ*_c_ approaches zero for small odd *m* (gain and loss defects close to the chain boundary). The localized states are non-$\mathcal{P}\mathcal{T}$-symmetric except for the two degenerate zero edge states, which can be composed into $\mathcal{P}\mathcal{T}$-symmetric form; and the real valued bound states appear when defects are at the chain center. For even *m*, the edge states are unaffected; for odd *m*, the edge states break the $\mathcal{P}\mathcal{T}$ symmetry when *m* is small (defects near the chain boundary). The $\mathcal{P}\mathcal{T}$ symmetry is fragile to nonzero non-Hermiticity; when *m* is large (defects near the chain center), the edge states are still unaffected because the probabilities of edge states decayed to zero at the locations of defects pair. In Fig. 4a, *γ*_c_ is no longer approaching 0 for odd *m* > 30 and monotonously increases as location *m* when *m* > 40. These all reflect that the influence of defects pair on the edge states is negligible and the two edge states energies are real and still being zero. The bound states appear in conjugation pairs, being non-degenerate; the bound states probabilities localize around each impurity. $\mathcal{P}\mathcal{T}$ symmetry is thus fragile to the bound states. An exception is that when the defects are at the chain center (*m* = 50), $\mathcal{P}\mathcal{T}$-symmetric bound states can appear in topologically nontrivial phase for *γ* < *γ*_c_ (in exact $\mathcal{P}\mathcal{T}$-symmetric phase). Figure 4b depicts the contours of *γ*_c_ at different location *m* as function of *θ* in full region of *θ* ∈ [−*π*, *π*]. At *m* = 1, *γ*_c_ maximum equals to 1 around |*θ*| = *π*/2 and shapely changes to zero in |*θ*| < *π*/2, where topologically nontrivial edge states appear. This is because that the edge states are fragile to the on-site non-Hermitian gain and loss. Affected by the edge states, the shape change of *γ*_c_ occurs near |*θ*| = *π*/2 at odd *m* for defects near the chain boundary. The influence of edge states vanishes for defects near the chain center and the bulk states induce $\mathcal{P}\mathcal{T}$ symmetry phase transition. Notably, *γ*_c_ increases dramatically at *m* = 50 in comparison with other cases. *γ*_c_ increases from 1 − Δ = 1/2 at |*θ*| = *π* to 1 + Δ = 3/2 at |*θ*| = 0. This large $\mathcal{P}\mathcal{T}$ transition threshold implies the $\mathcal{P}\mathcal{T}$-symmetric bound states may appear.

### The $\mathcal{P}\mathcal{T}$-symmetric bound states

In a large *N* system, the bound states located in the chain center have amplitude decayed to zero at the chain boundaries. At *m* = *n*, the bound states are analytically calculated. The eigenenergy is *E* = (*t*_1_ *χ* + *t*_2_)cos*ϕ*, where *sinϕ* = −*γ*/*t*_2_, *χ* is the decay factor that depends on the chain configuration and the inhomogeneous couplings. The eigenenergy is real for |*γ*/*t*_2_| ≤ 1. For even *n*, *t*_1_ = 1 − Δcos*θ*, *t*_2_ = 1 + Δcos*θ*; for odd *n*, *t*_1_ = 1 + Δcos*θ*, *t*_2_ = 1 − Δcos*θ*.

**Figure 5:**
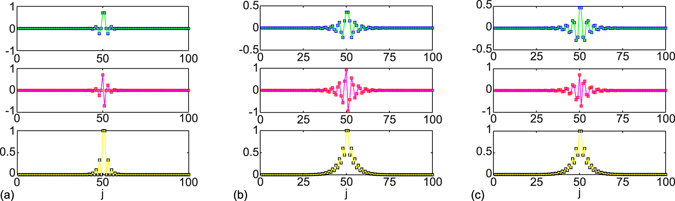
The $\mathcal{P}\mathcal{T}$-symmetric bound states. (**a**) The coalesced $\mathcal{P}\mathcal{T}$-symmetric bound states with *E* = 0 for *γ* = 3/2. One pair of $\mathcal{P}\mathcal{T}$-symmetric bound states are shown for *γ* = 1 in (**b, c**). (**b**) $E=2/\sqrt{5}$, (**c**) $E=-{\rm{2/}}\sqrt{5}$. The upper, middle, and lower panels are the real, imaginary and the absolute values of the wave function amplitude. The square markers represent the numerical results for *N* = 100, the solid lines are the analytical results for *N* → ∞. Other parameters are *m* = 50, *θ* = 0, Δ = 1/2.

Previously, the topologically protected $\mathcal{P}\mathcal{T}$-symmetric zero modes were demonstrated in non-Hermitian SSH chains, the SSH chains globally possess balanced gain and loss in each dimerized unit cell; the topologically protected zero modes are interface states that induced by the coupling disorder in the SSH chain centre and the global loss^48, 49, 51^. The interface states are confined to the passive sites with vanishing probability distributions on the lossy sites^49^, and spread on both sublattices^51^. In our SSH chain with one pair of balanced gain and loss at the centre, the existed $\mathcal{P}\mathcal{T}$-symmetric bound states are different. *The* $\mathcal{P}\mathcal{T}$ *-symmetric bound states are induced by the non-Hermitian local defects and significantly enhance the* $\mathcal{P}\mathcal{T}$ *transition threshold*. The two bound states energies are symmetric about zero energy and approach zero as the non-Hermiticity increases. At the $\mathcal{P}\mathcal{T}$ transition threshold, the bound states coalesce to the $\mathcal{P}\mathcal{T}$-symmetric zero mode, the zero mode is defective and topologically protected by the band gap^34^. The $\mathcal{P}\mathcal{T}$-symmetric zero mode still differs with that found at the interface between two topologically distinct $\mathcal{P}\mathcal{T}$-symmetric lattices induced by the coupling disorder^48, 49^. The coalesced $\mathcal{P}\mathcal{T}$-symmetric zero mode probability vanishes on every other site of the left-half chain and the right-half chain, respectively. The two $\mathcal{P}\mathcal{T}$-symmetric bound states are composed by the edge state localized on the right edge *n* of the left half chain and the edge state localized on the left edge *n* + 1 of the right half chain. When the coupling strength between the neighbours at chain center (sites *n* and *n* + 1) is stronger in the inhomogeneous couplings, the topologically protected zero mode appears at *γ* = *max*(*t*_1_, *t*_2_). The wave function contributions of the on-site defects ±*iγ* and the coupling between the neighbour sites *n* and *n* + 1 cancel each other, they mimic a free-like boundary except for *ψ*_*n*_ = *iψ*_*n*+1_ (*ψ*_*j*_ represents the wave function amplitude for site *j*). The wave function amplitude stepped decays in form of *χ* ^*l*^, where *l* is the dimerized unit cell index. The decay factor *χ* = −*t*_1_/*t*_2_ < 1 for even *n* (*χ* = −*t*_2_/*t*_1_ < 1 for odd *n*). In Fig. 5, the $\mathcal{P}\mathcal{T}$-symmetric bound states are depicted in topologically nontrivial phase at *θ* = 0. The real parts (upper panels) of the bound states are even functions of position while the imaginary parts (middle panels) of the bound states are odd functions of position. In this case (*t*_1_ = 1/2, *t*_2_ = 3/2), the $\mathcal{P}\mathcal{T}$ transition threshold is at *γ* = 3/2 = *t*_2_, the $\mathcal{P}\mathcal{T}$-symmetric bound states coalesce and turn to the topologically protected zero mode; we depicted it in Fig. 5a, the probability distribution vanishes for every other site of the left-half chain and the right-half chain, respectively. This topological zero mode differs with that found in the SSH chain with loss in each unit cell due to the distinct interface at the chain centre^48, 49^.

For |*γ*/*t*_2_| ≠ 1, the decay factor *χ* is

2 $$\chi =\frac{1}{2}\frac{{t}_{2}\,{sin}^{2}\varphi }{{t}_{1}\,{cos}^{2}\varphi }(1-\sqrt{1+{(\frac{{t}_{1}{cos}^{2}\varphi }{{t}_{2}{sin}^{2}\varphi })}^{2}\frac{4}{\,{cos}^{2}\varphi }}).$$

We choose the amplitude in the chain center |*ψ*_*n*+1_| = 1 instead of renormalized the wave function for convenience. In this situation, *ψ*_*n*+1_ = *e* ^*iϕ*/2^. The amplitude is [*ψ*_*n*+1_, *ψ*_*n*+2_, *ψ*_*n*+3_, *ψ*_*n*+4_, …] = *e* ^*iϕ*/2^[1, *χ* cos*ϕ*, *χ*, *χ* ^2^ cos*ϕ*, …], the bound states are not confined to one sublattice similar as the previously found zero mode^49^; and the distributions of the $\mathcal{P}\mathcal{T}$-symmetric bound states distinct at the centre due to the lattice difference^51^. For *γ* = 1 under *t*_1_ = 1/2, *t*_2_ = 3/2, we have $cos\,\varphi =\pm \sqrt{5}/3$; the corresponding decay factors for the two $\mathcal{P}\mathcal{T}$ symmetric bound states are both *χ* = −0.6 and the energies of the bound states are $E=\pm {\rm{2/}}\sqrt{5}$. The two $\mathcal{P}\mathcal{T}$-symmetric bound states are depicted in Fig. 5b,c. Notably, they have identical probability distributions.

The wave function of the $\mathcal{P}\mathcal{T}$-symmetric bound states at even *n* is

3 $${\psi }_{j}={e}^{i{\sigma }_{j}\varphi /2}\{\frac{1+\chi \,cos\,\varphi }{2}+{(-1)}^{[{\sigma }_{j}(j-{n}_{c})]}\frac{1+\chi \,cos\,\varphi }{2}\}{\chi }^{[{\sigma }_{j}(j-{n}_{c})/2]},$$

where *n*_c_ = (*N* + 1)/2, the power exponent [*σ*_*j*_(*j* − *n*_c_)] of (−1) in equation 3 represents the integer part of *σ*_*j*_(*j* − *n*_c_) and *σ*_*j*_ is a sign function defined as *σ*_*j*_ = sgn(*j* − *n*_c_). At odd *n* case, the expression of *ψ*_*j*_ is still valid; however, the bound states are not real valued and $\mathcal{P}\mathcal{T}$-symmetric bound states vanish. The values *ϕ* for the bound states with complex eigenvalues are ${\varphi }_{1}={sin}^{-1}(-\gamma /{t}_{2})$, ${\varphi }_{2}=-{sin}^{-1}(-\gamma /{t}_{2})$, ${\varphi }_{3}=\pi -{sin}^{-1}(-\gamma /{t}_{2})$, and ${\varphi }_{4}=-\pi +{sin}^{-1}(-\gamma /{t}_{2})$; the corresponding decay factors *χ* and the wave function of bound states can be obtained from equations 2 and 3.

## Conclusion

We have studied a pair of balance gain and loss defects in a non-Hermitian SSH chain, the influence differs significantly as the $\mathcal{P}\mathcal{T}$-symmetric defects locations. The $\mathcal{P}\mathcal{T}$ transition threshold has been investigated, the number of broken energy levels at maximum increases as the defects close to the chain center in the broken $\mathcal{P}\mathcal{T}$-symmetric phase; for the defects at the chain center, all energy levels break the $\mathcal{P}\mathcal{T}$ symmetry simultaneously in topologically trivial phase, but two edge states are free from $\mathcal{P}\mathcal{T}$ symmetry breaking in topologically nontrivial phase. When the defects are near the chain boundaries, the edge states in topologically nontrivial phase break the $\mathcal{P}\mathcal{T}$ symmetry if defects are at the sites with nonzero edge states distribution probabilities; the $\mathcal{P}\mathcal{T}$ symmetry breaking is caused by the extended states at weak non-Hermiticity or by the bound states at strong non-Hermiticity. The bound states probabilities are localized at the defects and decay exponentially, thus are $\mathcal{P}\mathcal{T}$-symmetric breaking; however, the $\mathcal{P}\mathcal{T}$-symmetric bound states can be formed when the defects are at the SSH chain center, where the gain and loss are the nearest neighbors. Therefore, the $\mathcal{P}\mathcal{T}$ transition threshold in this situation increases significantly, which is the largest and equals to the weak inhomogeneous coupling. The $\mathcal{P}\mathcal{T}$-symmetric bound states are the topologically protected coalesced zero mode at the $\mathcal{P}\mathcal{T}$ transition threshold.
